# Real-World Sex Differences in Response to Treatment with Glucagon-like Peptide-1 Receptor Agonists: Analysis of Single-Center Outpatient Case Series

**DOI:** 10.3390/medicina61081343

**Published:** 2025-07-25

**Authors:** Georgeta Victoria Inceu, Anca-Elena Crăciun, Dana Mihaela Ciobanu, Antonia Berchisan, Adriana Fodor, Cornelia Bala, Gabriela Roman, Adriana Rusu

**Affiliations:** 1 Department of Diabetes and Nutrition Diseases, “Iuliu Hațieganu” University of Medicine and Pharmacy, 2-4 Clinicilor St., 400006 Cluj-Napoca, Romania; inceu.victoria@umfcluj.ro (G.V.I.); dana.ciobanu@umfcluj.ro (D.M.C.); adriana.fodor@umfcluj.ro (A.F.); cbala@umfcluj.ro (C.B.); groman@umfcluj.ro (G.R.); adriana.rusu@umfcluj.ro (A.R.); 2 Department of Diabetes, Nutrition and Metabolic Diseases, Emergency Clinical County Hospital Cluj, 400006 Cluj-Napoca, Romania; berchisanantonia@gmail.com; 3 2nd Department, Faculty of Nursing and Health Sciences, “Iuliu Hațieganu” University of Medicine and Pharmacy, 4 Louis Pasteur Street, 400349 Cluj-Napoca, Romania

**Keywords:** type 2 diabetes, glucagon-like peptide-1, sex differences, weight management, glycemic control

## Abstract

*Background and Objectives*: Type 2 diabetes (T2D) is a global health burden with increasing prevalence, necessitating effective management strategies. Glucagon-like peptide-1 receptor agonists (GLP-1 RAs) have emerged as beneficial therapies, promoting both glycemic control and weight loss, yet real-world data on sex differences in response are limited. This study aimed to investigate sex-based differences in glycemic and weight outcomes, as well as adverse effects, in T2D patients treated with GLP-1 RAs at a single diabetes center. *Materials and Methods*: In this retrospective analysis, 114 patients (58.8% men) with T2D who were initiated on GLP-1 RA therapy between 2015 and 2023 were evaluated. Data on HbA1c, BMI, and adverse events were collected at baseline and 3, 6, and 12 months post-treatment initiation. *Results*: Our findings indicated a statistically significant HbA1c reduction (from 8.6% at GLP-1 RA initiation to 6.9% at 12 months in men (*p* < 0.001) and from 8.4% at initiation to 7.0% at 12 months after GLP-1 RA initiation (*p* < 0.001) in women). By 12 months, a significantly greater proportion of women compared to men achieved ≥ 5% (51.1% vs. 28.4%, *p* = 0.019) and ≥10% weight loss (29.8% vs. 9.0%, *p* = 0.006), with both differences reaching statistical significance. A statistically significant difference in weight loss in mean weight change and percent weight change in men vs. women was observed from month 6 of therapy. *Conclusions*: These findings underscore the effectiveness of GLP-1 RAs in improving glycemic control and weight loss in a real-world setting and suggest that women may experience greater weight reduction. Understanding these differences could inform personalized treatment strategies for optimized outcomes in T2D management.

## 1. Introduction

Type 2 diabetes (T2D) is a chronic metabolic disorder that represents a significant global health burden [1]. Chronic hyperglycemia leads to severe complications, including cardiovascular disease, kidney failure, neuropathy, and retinopathy. As the prevalence of T2D continues to rise, particularly in middle- and high-income countries, effective long-term management strategies are needed to reduce morbidity and improve patient outcomes [2]. The primary goals in T2D management are to achieve and maintain optimal glycemic control, reduce body weight in overweight or obese patients, and prevent complications associated with prolonged hyperglycemia [3]. Lifestyle modifications and traditional therapeutic approaches, like metformin, sulfonylureas, and insulin, often fail to achieve adequate glycemic control without adverse effects such as weight gain and hypoglycemia. In recent years, we have witnessed a paradigm shift in the treatment of T2D. Innovative classes of antihyperglycemic agents have been developed, including sodium–glucose transporters 2 (SGLT2) inhibitors and glucagon-like peptide-1 receptor agonists (GLP-1 RAs), which not only improve glycemic control without promoting weight gain but also offer additional cardiorenal benefits. These advantages have positioned these agents as the mainstay of treatment for patients with T2D [4].

GLP-1 RAs have emerged as an important class of antidiabetic drugs that offer both glucose-lowering and weight-reduction benefits. These agents mimic the action of endogenous GLP-1, an incretin hormone that stimulates insulin secretion, inhibits glucagon release, slows gastric emptying, and promotes satiety [5]. Consequently, GLP-1 RAs not only improve glycemic control but also lead to clinically significant weight loss, making them particularly valuable for overweight and obese patients with T2D [6,7]. In addition, multiple cardiovascular outcomes trials have shown significant reductions in major adverse cardiovascular events (MACEs), like myocardial infarction, stroke, or cardiovascular mortality, as well as reduction in hospitalizations for heart failure and slower progression of diabetic chronic kidney diseases among patients treated with GLP-1 RAs [8]. Several GLP-1 RAs have been approved for use, including exenatide, liraglutide, dulaglutide, and semaglutide, each with distinct pharmacokinetic profiles and administration regimens.

While the efficacy of GLP-1 RAs in lowering hemoglobin A1c (HbA1c) and promoting weight loss has been well documented in clinical trials [9,10], there is a large variability in response. While some patients improve both HbA1c and weight, others may benefit from one of these or none. Moreover, little is known about how these benefits translate into real-world clinical practice. Furthermore, potential differences in treatment response based on patient characteristics, such as sex, have garnered increasing interest. Understanding whether men and women respond differently to GLP-1 RAs in terms of glycemic control, weight loss, and adverse effects is essential for tailoring personalized treatment strategies and enhancing clinical outcomes.

Sex-based differences in response to diabetes therapies are biologically plausible due to variations in body composition, hormonal profiles, and pharmacokinetics between men and women [11]. Prior studies have suggested potential sex differences in response to GLP-1 RA therapy, particularly in terms of glycemic control, weight reduction, and adverse drug reactions, but real-world data on this topic are limited and remain to be verified [12]. In a large-scale retrospective pooled analysis of patients receiving exenatide twice daily, HbA1c reduction appeared to be irrespective of sex but highly dependent on baseline HbA1c values [13]. Another pooled analysis of clinical trials that evaluated the use of dulaglutide in people with diabetes concluded that sex did not influence the effect of dulaglutide in reducing HbA1c level [14]. By contrast, the efficacy of GLP-1 RAs in reducing HbA1c was found to be superior in females vs. males in several other studies [15,16]. Weight loss is clearly one of the main benefits of GLP-1 RA treatment. In this regard, a consistent body of evidence from the literature suggests that females consistently benefited from greater weight loss when compared with males [14,17].

GLP-1 RAs have a generally favorable safety profile. The most common side effects are gastrointestinal, including nausea, vomiting, diarrhea, and constipation. These side effects tend to occur during the initial phase of treatment and often diminish over time. Serious adverse effects are rare but may include pancreatitis and gallbladder-related issues like gallstones. Other concerns include an increased risk of thyroid C-cell tumors, although this has been seen mainly in animal studies, and human data have not confirmed this risk [18]. Most studies focusing on safety profile of GLP-1 RAs have indicated that women may be more susceptible to gastrointestinal side effects compared with men, suggesting that female sex could be an independent risk factor for adverse events in GLP-1 RA therapy [12,19,20].

This study aims to address this knowledge gap by conducting a retrospective analysis of T2D patients treated with GLP-1 RAs from a single diabetes center. The study focuses on real-world sex differences in the efficacy of GLP-1 RAs for improving glycemic control and inducing weight loss, as well as the incidence of adverse drug reactions. These findings could have important implications for personalized diabetes care and may help clinicians make more informed decisions regarding the selection and management of GLP-1 RAs in their patients with T2D.

## 2. Materials and Methods

For this retrospective study, data were collected from the outpatient records of the diabetes department of a county emergency hospital. We included adult T2D patients in whom the therapy with a GLP-1 RA was initiated between June 2015 and July 2023 and who had at least 1 assessment at 3, 6, or 12 months after therapy initiation. Patients with type 1 diabetes, specific forms of diabetes, or patients <18 years of age were excluded. The study protocol was approved by the Institutional Ethics Committee of “Iuliu Hatieganu” University of Medicine and Pharmacy Cluj-Napoca, Romania (No.47/10.03.2016). No specific informed consent was deemed necessary due to its retrospective design.

For patients newly initiated on GLP-1 RAs, data was collected from patients’ files: sex, age, residency, diabetes duration, previous diagnosis of chronic diabetes complications (diabetic retinopathy, neuropathy, and chronic kidney disease), atherosclerotic cardiovascular diseases (coronary heart disease, stroke, peripheral arterial disease), GLP-1 RA name, HbA1c (%), body weight (kg), and height (m). At each subsequent evaluation at 3, 6, and 12 months after the GLP-1 RA initiation, we collected the value of HbA1c, weight, therapy discontinuation and the reason for it, and adverse drug reactions which were deemed as related to the GLP-1 RAs by the physician. BMI was calculated as weight (kg)/height (m)^2^.

Early responders were considered patients with more than 5% weight loss [21] and more than 1% HbA1c reduction after 3–6 months of therapy [22]. Non-responders for weight loss were defined as patients with a weight loss < 5% at all timepoints [23,24]. Non-responders for glycemic management were considered patients with no improvement or increase in the HbA1c if the baseline value was <7% or reduction of less than 0.5% of HbA1c if the starting value was over 7% [25].

For the research presented in the manuscript we have collected all adverse drug reactions as documented in the patients’ medical records. Specifically, only those adverse events that the physician explicitly recorded as being temporally associated with the initiation or continuation of GLP-1 RA therapy and considered likely to be related to the medication (based on clinical presentation and exclusion of alternative causes) were collected and included in the analysis.

### Statistical Analysis

No sample size calculation was performed and a convenience sample of 114 patients fulfilling the inclusion criteria and without any exclusion criterion was chosen. Statistical analysis was performed with IBM SPSS Statistics for Windows, Version 26.0 (Armonk, NY: IBM Corp). Changes from baseline were calculated for each individual patient and then summarized for men and women. Data were presented as mean ± standard deviation for continuous variables and n (%) for categorical variables. Variables in men and women were compared using *t*-tests and chi-square tests according to the type of variables included. Mean HbA1c and BMI at each timepoint were compared to baseline (GLP-1 RA initiation) using the paired-sample *t*-test. Also, for various timepoints, change in mean weight and HbA1c from baseline were compared between men and women using the Mann–Whitney U test. The effect size was calculated using Cohen’s d (95% confidence interval (CI)) and Cramer’s V for the difference between men and women in weight and HbA1c change.

A linear regression analysis performed separately in men and women and adjusted for age and diabetes duration at GLP-1 RA initiation and comorbidities was used to explore the effect of initial weight and HbA1c on the weight change and HbA1c change observed at 3, 6, and 12 months. We have chosen these variables for the adjustment of the regression models based on previous research which showed that weight loss and HbA1c change after GLP-1 RA initiation might be influenced by several factors including age and the presence of chronic microvascular complications and cardiovascular disease [26,27,28]. Mechanisms hypothesized to be involved were associated medication for comorbidities (such as beta blockers), effect on appetite and satiety signals, higher risk of gastrointestinal adverse drug reactions, and lower medication adherence in those with diabetic neuropathy [27,29]. Also, the presence of existing chronic kidney disease and cardiovascular disease may influence the choice of GLP-1 RAs over other drug classes due to their additional renal and cardiovascular benefits [28,30].

As the sample sizes for several GLP-1 RAs included in the analysis were small, we chose to perform an exploratory analysis by GLP-1 RA type. We compared the weight and HbA1c changes in men and in women and each timepoint by GLP-1 RA type using the Mann–Whitney U test.

A *p*-value < 0.05 was considered statistically significant.

## 3. Results

### 3.1. Participant Characteristics

In total, 114 patients with T2D (58.8% men) with at least one evaluation available within 12 months following GLP-1 RA initiation were included in this analysis. Of the included patients, 88 had an evaluation at 3 months, 95 at 6 months, and 97 at 12 months after the GLP-1 RA initiation. Diabetes duration at GLP-1 RA initiation was 8.9 years and the most frequent chronic microvascular complication was diabetic neuropathy (reported in 43.0% of the patients). Mean HbA1c at GLP-1 RA initiation was 8.5% (minimum 5.8%, maximum 14.1%), similar in men and women (*p* = 0.595). Mean BMI was 35.1 kg/m^2^ (minimum 24.1 kg/m^2^, maximum 56.5 kg/m^2^), with no statistically significant differences between men and women (*p* = 0.060; Table 1). Regarding GLP-1 RA type, patients were prescribed daily or weekly exenatide (5.0%), oral or injectable semaglutide (68.0%), lixisenatide (2.0%), dulaglutide (22.0%), or liraglutide (3.0%). GLP-1 RA was administered as an add-on therapy to other glucose-lowering medications, most commonly metformin (97.4%), followed by sulphonylureas (17.5%), SGLT-2 inhibitors (13.2%), basal insulin (29.8%), and rapid-acting insulin (2.6%). There were no statistically significant differences between sexes regarding the associated antihyperglycemic drugs.

### 3.2. Efficacy in Improving Glycemic Control

In the overall study population, the mean HbA1c decreased from 8.5% at GLP-1 RA initiation to 7.2% at 3 months, 7.1% at 6 months, and 7.0% at 12 months (*p* < 0.001 for all timepoints vs. GLP-1 RA initiation). In men, HbA1c decreased from 8.6% at GLP-1 RA initiation to 6.9% at 12 months (*p* < 0.001) and in women from 8.4% at initiation to 7.0% at 12 months (*p* < 0.001; Supplementary Figure S1).

**Figure 1:**
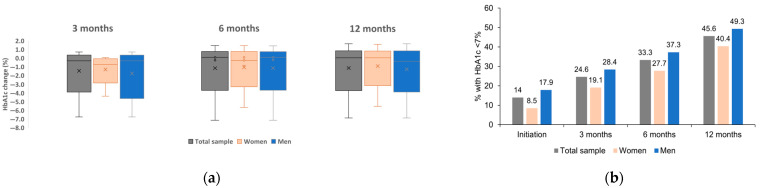
Mean HbA1c change by sex (**a**) and percentage of men and women with an HbA1c < 7% at 3, 6, and 12 months (**b**). No statistically significant difference in HbA1c between women and men was observed at any timepoint (*p* > 0.05; Mann–Whitney U test). A statistically significant higher percentage of men achieved and HbA1c < 7% at 3 months (*p* = 0.029, chi-square test).

The mean HbA1c change from GLP-1 RA initiation was −1.4% at 3 and 6 months and −1.5% at 12 months. There was no statistically significant difference in mean HbA1c change between men and women at any timepoints after GLP-1 RA initiation (*p* = 0.229 at 3 months, 0.207 at 6 months, and 0.355 at 12 months). In women, the mean HbA1c change was −1% at 3 months, −1.2% at 6 months, and −1.4% at 12 months, while in men HbA1c change was −1.7% at 3 months, −1.5% at 6 months, and −1.6 at 12 months (Figure 1a). The effect size for sex was moderate for the difference at 3 months with d = −0.388 (95%CI: −0.958; 0.186) and small for the difference in the mean HbA1c change between men and women at 6 and 12 months (d = −0.191 (95%CI: −0.660; 0.279) and d = −0.070 (95%CI: −0.498; 0.359), respectively).

The percentage of patients with HbA1c < 7% increased from 14.0% at GLP-1 RA initiation to 45.6% at 12 months. A statistically significantly higher percentage of men compared to women achieved an HbA1c < 7% at 3 months (*p* = 0.029). A similar percentage of men and women achieved an HbA1c < 7% at 6 and 12 months (*p* = 0.213 and 0.355, respectively for the comparison between women and men; Figure 1b). The effect size (Cramer’s V) of sex was moderate (0.305) for the difference at 3 months and small for 6 and 12 months (0.146 and 0.097, respectively).

**Figure 2:**
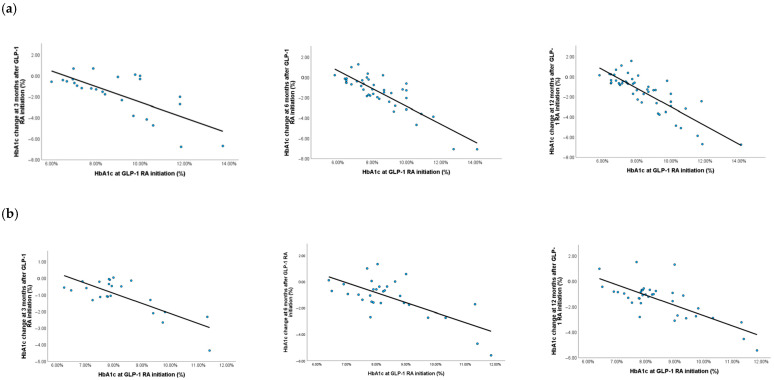
Linear association of HbA1c change at 3, 6, and 12 months with HbA1c at GLP-1 RA initiation in men (**a**) and women (**b**).

By linear regression analysis adjusted for age and diabetes duration at GLP-1 RA initiation, diabetes chronic complications, and cardiovascular diseases, we explored whether HbA1c change during therapy was influenced by HbA1c level at baseline. In both men and women, the value of HbA1c at GLP-1 RA initiation was a predictor of HbA1c decrease at all timepoints, with a higher decrease observed in those with a higher HbA1c at baseline. In men there was a negative association of HbA1c at baseline with HbA1c change at all timepoints, with β = −0.793, *p* = 0.004 at 3 months; β = −0.708, *p* <0.001 at 6 months; β = −0.828, *p* <0.001 at 12 months. A similar negative association was observed in women with β = −0.662, *p* = 0.006 at 3 months; β = −0.691, *p* = 0.010 at 6 months; β = −0.702, *p* = 0.001 at 12 months (Figure 2).

An exploratory analysis of HbA1c changes associated with various GLP-1 RAs by sex showed no statistically significant differences between women and men with either molecule at all timepoints (*p* > 0.1 for the comparison between women and men at each timepoint).

### 3.3. Weight Loss Efficacy

In the whole sample, mean BMI decreased from 35.1 kg/m^2^ at GLP-1 RA initiation to 34.1 kg/m^2^ at 3 months, 32.6 kg/m^2^ at 6 months, and 32.3 kg/m^2^ at 12 months after GLP-1 RA initiation (*p* < 0.001 for all comparisons at each timepoint vs. GLP-1 RA initiation). A statistically significant BMI reduction from baseline was observed in both men and women at all timepoints analyzed (*p* < 0.001 for all comparisons at each timepoint vs. GLP-1 RA initiation in both men and women; Supplementary Figure S2).

**Figure 3:**
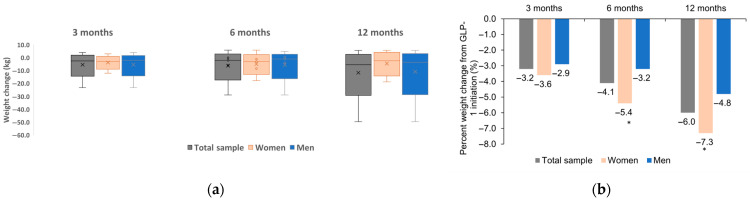
Mean (**a**) and percent (**b**) weight change at 3, 6, and 12 months. * denotes statistically significant difference of percent weight change in men vs. women at 6 months (*p* = 0.026) and 12 months (*p* = 0.011). At each timepoint men and women were compared using the Mann–Whitney U test.

No statistically significant difference between men and women was observed in weight change from baseline at 3 months (*p* = 0.233). At 6 and 12 months, women lost statistically significantly more weight as compared to men (*p* = 0.047 and *p* = 0.038; Figure 3a). In terms of effect size (Cohen’s d) for the interaction of sex with weight change it was small for 3 months (d = 0.093; 95%CI: −0.357; 0.543) and moderate for 6 months (d = 0.357; 95%CI: −0.119; 0.831) and 12 months (d = 0.224; 95%CI: −0.220; 0.666).

Similarly, the percentage change in body weight was statistically similar at 3 months between men and women (*p* = 0.211) and statistically significantly higher in women than in men at 6 months (*p* = 0.026) and at 12 months (*p* = 0.011; Figure 3b). The effect size (Cohen’s d) of sex was small at 3 months (d = 0.175; 95%CI: −0.276; 0.625) and moderate at 6 months (d = 0.473; 95%CI: −0.006; 0.949) and 12 months (d = 0.395; 95%CI: −0.052; 0.840).

In the overall sample the proportion of patients who achieved at least 5% weight loss was 22.8% at 3 months and increased to 25.4% at 6 months and 37.7% at 12 months after the GLP-1 RA initiation. By sex, the difference between the percentage of men and women who achieved at least 5% weight loss was not statistically significant at 3 and 6 months (22.4% vs. 23.4%, *p* = 0.650 and 20.9% vs. 31.9%, *p* = 0.104). However, at 12 months, a statistically significantly higher percentage of women than men achieved a weight reduction of at least 5% body weight from baseline (51.1% vs. 28.4%, *p* = 0.019). The effect size (Cramer’s V) of sex was small for all timepoints (0.051 at 3 months; 0.192 at 6 months; 0.262 at 12 months).

A reduction of more than 10% of initial body weight was achieved by 2.6% of the whole sample analyzed at 3 months, 5.3% at 6 months, and 17.5% at 12 months after the GLP-1 RA initiation. The difference between the percentage of men and women who had at least 10% weight loss was not statistically significant at 3 and 6 months (3.0% vs. 2.1%, *p* = 0.844 and 3.0% vs. 8.5%, *p* = 0.169, respectively). At 12 months, a statistically significantly higher percentage of women than men achieved a weight reduction of at least 10% body weight from baseline (29.8% vs. 9.0%, *p* = 0.006). The effect size (Cramer’s V) of sex was small for 3 and 6 months (0.022 at 3 months; 0.162 at 6 months) and moderate for 12 months (0.306 at 12 months).

**Figure 4:**
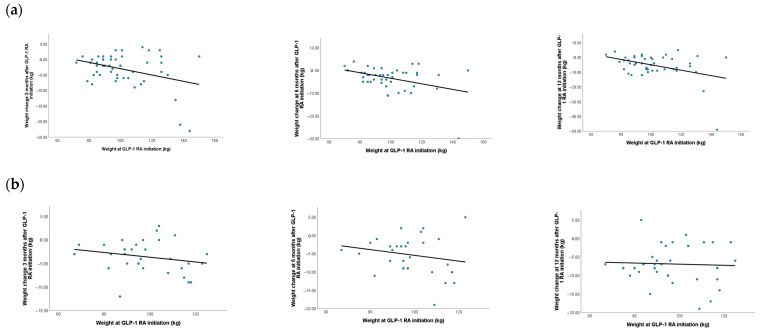
Linear association of weight change at 3, 6, and 12 months with weight at GLP-1 RA initiation in men (**a**) and women (**b**).

By linear regression analysis adjusted for age and diabetes duration at GLP-1 RA initiation, chronic microvascular complications, and cardiovascular diseases, we explored whether weight at GLP-1 RA initiation was correlated with weight change during therapy. In men we observed an inverse linear association of initial weight with weight change at 6 and 12 months after GLP-1 RA initiation (β = −0.428, *p* = 0.042 at 6 months and β = −0.460, *p* = 0.031 at 12 months), with greater decrease in weight in those with higher weight at baseline. No association of weight change with weight at GLP-1 RA initiation was observed in men at 3 months (*p* = 0.121) or in women at any timepoint (*p* = 0.859 at 3 months; *p* = 0.586 at 6 months; and *p* = 0.881 at 12 months; Figure 4).

We have also performed an exploratory analysis on the weight change associated with various GLP-1 RAs by sex. We have observed a greater weight decrease in women than in men with semaglutide at 6 months (−6.5 kg. vs. −3.9 kg, *p* = 0.030 for weight change in women vs. men comparison) and 12 months (−8.9 kg vs. −6.3 kg, *p* = 0.017 for weight change in women vs. men comparison, Mann–Whitney U test). No statistically significant difference in weight change between men and women was observed for exenatide, lixisenatide, dulaglutide, and liraglutide (*p* > 0.1 for weight change in women vs. men comparison at each timepoint).

### 3.4. Early Responders and Non-Responders

Of the patients, 26.8% meet the criteria of early responders regarding the glycemic and weight loss parameters (>5% weight loss and >1% HbA1c reduction after 3–6 months of therapy).

Non-responders for weight loss (defined as weight loss < 5%) were 67.5% in the first 3 months, 59.7% at 6 months, and 46.3% after one year of GLP-1RA therapy. Non-responders for glycemic management at 3 months, 6 months, and 1 year of GLP-1RA therapy were 29.2%, 16.7%, and 18.6%, respectively. The proportions of non-responders for both glycemic and weight parameters were 16.3% after 3 months, 12% after 6 months, and 10.4% after 12 months. Due to small numbers, we did not perform further analysis between groups.

### 3.5. Adverse Drug Reactions

Adverse drug reactions were reported in 21 patients (18.4%) and led to treatment discontinuation in 8 patients (7% of the total sample). There was no statistically significant difference between men and women in the incidence of adverse drug reaction (16.4% vs. 21.3%, *p* = 0.166). The most frequent adverse drug reactions in both sexes were gastrointestinal events, accounting for 63.0% of the reactions in men and 90.0% of the reactions in women (*p* = 0.395).

## 4. Discussion

In this retrospective cohort study involving consecutive patients with T2DM, we analyzed sex differences in response to treatment with GLP-1 RAs in the first year of follow-up. There was no statistically significant difference between men and women in the mean HbA1c change after GLP-1 RA initiation and in both sexes the value of HbA1c at GLP-1 RA initiation was a predictor of HbA1c decrease, with a higher decrease observed in those with a higher HbA1c at baseline. Regarding the weight loss response after initiation of GLP-1 RA therapy, there was no statistically significant difference between men and women in the first 6 months of therapy, but at 1 year, the percentages of women who achieved more than 5% or 10% weight loss were statistically significantly higher than in men. In a linear regression analysis adjusted for age, diabetes duration, chronic microvascular complications, and cardiovascular disease, we observed in men a statistically significant inverse association between baseline body weight and weight change at 6 and 12 months following GLP-1 RA initiation. This association was not observed at 3 months in men nor at any timepoint in women. Subanalysis of the weight loss response by GLP-1 RA type showed a greater weight decrease in women than in men with semaglutide at 6 months, but no statistically significant difference in weight change between men and women was observed for exenatide, lixisenatide, dulaglutide, and liraglutide.

In our study population, GLP-1 RA therapy was initiated in patients with a long duration of diabetes, poor glycemic control, and obesity, with no baseline differences between sexes. These data were also observed in other real-world studies addressing GLP-1 RA therapy in type 2 diabetes [31,32]. GLP-1 RAs are comparable to basal insulin regarding the glycemic effect, especially long-acting agents (semaglutide and dulaglutide) [33]. Due to the efficacy in reducing HbA1c, accompanied by a weight loss effect, low risk of hypoglycemia, and ease of use (simple titration plan and once-daily or even once-weekly administration), the ADA/EASD Guideline recommends to preferentially use GLP-1 RAs in T2D patients before insulin therapy [3,4]. Of note is that we had in our study patients with HbA1c in the target range, who received GLP-1 RAs for cardiovascular protection, according to recent guideline recommendations to initiate GLP-1 RAs regardless of glycemic control in patients with high and very high cardiovascular risk [3]. Over 90% of our patients received semaglutide or dulaglutide, which have proven cardiovascular benefits. The GLP-1 RA class has expanded in recent years, and weekly injection or oral administration is preferred [34]. A recent published study by Piccini S et al. showed that discontinuation of GLP-1 RA therapy over time was associated with MACE risk in primary prevention in both sexes and only in men with previous CV events [35]. Another interesting observation concerns the sex distribution of participants in GLP-1 RA clinical trials. In trials focused on obesity, the proportion of female participants is higher, whereas in studies involving patients with type 2 diabetes, a higher percentage of male participants is typically observed, as was the case in our study [36].

The mean reduction in HbA1c during the first year following GLP-1 RA initiation was −1.5%. The maximum glycemic efficacy of the GLP-1 RAs, almost 90% of the effect, was observed in the first 3 months of therapy (−1.3% reduction of HbA1c), with slight improvements of the HbA1c over the next months of treatment (−0.2% supplementary reduction of HbA1c at month 12 versus month 3). Furthermore, Chen Y et al. recently showed that GLP-1 RAs improved time spent in range by T2D patients after 3–6 months of therapy versus OADs, with better results with once-weekly GLP-1 RAs versus once-daily GLP-1 RAs [37].

There was no statistically significant sex-related difference in HbA1c reduction in our cohort, and a similar proportion of men and women achieved an HbA1c level below 7% at 12 months. Comparable findings were reported in a pooled analysis of the AWARD-1 to AWARD-6 and AWARD-8 trials, where weekly administration of 1.5 mg of dulaglutide resulted in HbA1c reductions of −1.26% in men and −1.36% in women [14]. Also, subgroup analyses of patients treated with semaglutide revealed sex differences in glycemic response, but with no convergent results [38].

Regarding the weight loss effect of GLP-1 RA therapy, there was a significant and progressive decrease in BMI over the first 12 months of therapy. A statistically significantly higher percentage of women achieved, at 12 months, over 5% or over 10% weight loss compared with men. Cumulative evidence indicates that women experience greater weight loss effects than men. This interesting phenomenon was observed in studies carried out in a T2D population [17] and in people with obesity without T2D [39]. One explanation might be different exposures to drugs. The pharmacokinetic analysis of samples drawn during SCALE Obesity and Prediabetes and SCALE Diabetes studies showed a 32% higher exposure in women than men with the same body weight [40]. Observations from other studies showed that women experienced more gastrointestinal side effects than men and this may be indirectly associated with increased GLP-1 RA efficacy in women [12]. Although more frequent in women, gastrointestinal side effects did not reach statistical significance. The magnitude of glycemic and weight-related responses to GLP-1 RAs varies among patients, with not all individuals experiencing comparable therapeutic benefits. Regarding the glycemic effect, GLP-1 RAs demonstrate efficacy comparable to that of basal insulin. Their potency appears to increase with higher baseline HbA1c levels, and in patients with lower baseline HbA1c, GLP-1 RAs may outperform basal insulin—possibly due to suboptimal insulin titration driven by concerns over hypoglycemia [41]. One hypothesis for inter-individual variability of response might be linked to genetic variants. Dawed AY et al. recently published a genome-wide pharmacogenomic study of GLP-1 RAs and observed that some variants of the β-arrestin1 gene (ARBB1) and GLP-1 receptor gene (GLP1R) are associated with better glycemic response in the first 6 months of therapy [42].

The early glycemic and/or weight response observed in the first 3–6 months of GLP-1 RA therapy is associated with long-term persistence and adherence in patients with T2D [43]. One in four patients included in our study was an early responder, meaning more than 5% weight loss and more than 1% HbA1c reduction after 3–6 months of therapy. Regarding the weight loss effect, the variability in response is higher than variability in glycemic response and the current knowledge can only partially explain this observation [44]. There have been several studies investigating responders and non-responders regarding the weight loss effect. One study, conducted on 22 healthy subjects, showed that 11 participants were non-responders to exenatide infusion. Non-responders were defined as individuals exhibiting less than a 10% reduction in caloric intake following GLP-1 RA administration. Despite the lack of appetite suppression, non-responders demonstrated comparable glycemic reductions, suggesting a preserved pancreatic effect. Moreover, baseline characteristics and biomarkers did not account for the variability in treatment responsiveness. Finally, functional MRI analysis showed that responders had significantly higher hypothalamic connectivity than non-responders, suggesting that the weight effect of GLP-1 RAs is mediated by the responsiveness of the hypothalamus to the pharmacological agent [44].

In T2D patients a significant weight loss (more than 5% of the initial body weight) was observed only in one third of the patients, meaning that two thirds of the patients were non-responders to the weight loss effect [45]. In our study we observed that, after one year of therapy, one in two patients was a non-responder to the weight loss effect, but the percentage of non-responders decreased over the first year of therapy (from 67.5% at 3 months to 46.25% at 12 months). One in ten patients was a non-responder to both glycemic and weight loss effects after 12 months of GLP-1 RA therapy. In such cases, therapeutic adherence may also represent a contributing factor; however, a reliable method to assess compliance with GLP-1 RA therapy in clinical practice is lacking, unlike the indirect assessment available for SGLT2 inhibitors, such as the presence of glycosuria.

In our study the most frequent adverse events in both sexes were gastrointestinal ones, accounting for 63.0% of the reactions in men and 90.0% of the reactions in women. Nausea is reported in GLP-1 RA studies in up to 25% of the participants, followed by vomiting and diarrhea in up to 10% [33]. Gastrointestinal side effects are more prominent at the initiation of the drug or increments of the dose and their appearance/persistence in fasting states indicates that the mechanism is not related only to the effect on the gastro-intestinal tract but also to the central effect on specific brain areas. In our study the 7% of patients who stopped administration of GLP-1 RAs due to adverse drug reactions is comparable to the observations made in cardiovascular outcome trials, where proportions of patients discontinuing the study drug due to adverse events ranged from 4.5 to 13.2% [33]. There was no statistically significant difference in the percentage of men and women reporting adverse drug reactions (16.4% vs. 21.3%, *p* = 0.166).

This study has several limitations that should be considered when interpreting the findings. First, the retrospective design and relatively small sample size may limit the statistical power to detect subtle sex-related differences, particularly in subgroup analyses. Second, the single-center nature of the study may affect the applicability of our results to broader or more diverse populations. Another limitation is the lack of sample size and power calculation, which may influence the results observed and make it more difficult to detect true effects and reject a false null hypothesis. The study also spans an 8-year period (2015–2023), during which treatment guidelines, prescribing patterns, and the availability of GLP-1 RAs have evolved, potentially influencing treatment selection and outcomes. Additionally, our dataset did not include information on individual GLP-1 RA dosing or dose adjustments over time. Given that these agents are generally administered at fixed doses, women—who typically have lower body weight—may have received relatively higher doses per kilogram, possibly contributing to the greater weight loss observed.

Other limitations include the lack of standardized data on adherence or compliance with GLP-1 RA therapy, which may have influenced the magnitude of treatment effects. Furthermore, although we reported the most common classes of concomitant antihyperglycemic agents, we did not capture all concurrent medications, including those that could potentially affect weight or glycemic control. Lastly, unmeasured confounding factors—such as dietary patterns, physical activity, and socioeconomic status—were not available and may have contributed to the sex-related differences in treatment response.

## 5. Conclusions

This single-center retrospective analysis highlights significant findings regarding sex differences in response to GLP-1 RAs in real-world T2D management. Both men and women experienced improvements in glycemic control, with no substantial sex-related variation in HbA1c reduction. However, women achieved notably greater weight loss than men, particularly after 12 months of treatment. Although adverse drug reactions, primarily gastrointestinal, were slightly more frequent in women, no statistically significant sex differences were observed. These findings suggest that sex-specific considerations may enhance GLP-1 RA treatment strategies, promoting personalized, effective care for T2D patients.

## Figures and Tables

**Table 1:** Participant characteristics analyzed by sex.

	Total Sample *n* = 114	Women *n* = 47	Men *n* = 67	*p*-Value
Area of residence (urban) *n* (%)	93 (81.6%)	38 (80.9%)	55 (82.1%)	0.867
Age at GLP-1 RA initiation, years	60.4 ± 10.1	60.8 ± 10.0	60.2 ± 10.2	0.760
Body mass index at GLP-1 RA initiation, kg/m^2^	35.1 ± 6.0	36.4 ± 5.6	34.1 ± 6.1	0.060
Diabetes duration, years	8.9 ± 6.7	12.3 ± 6.2	10.0 ± 7.5	0.087
HbA1c at GLP-1 RA initiation, %	8.5 ± 1.7	8.4 ± 1.3	8.6 ± 1.9	0.595
Retinopathy, *n* (%)	13 (11.4%)	6 (12.8%)	7 (10.4%)	0.346
Diabetic neuropathy, *n* (%)	49 (43.0%)	22 (46.8%)	27 (40.3%)	0.760
Chronic kidney disease, *n* (%)	19 (16.7%)	2 (4.3%)	17 (25.4%)	0.007
Stroke, *n* (%)	7 (6.1%)	3 (6.4%)	4 (6.0%)	0.916
Peripheral artery disease, *n* (%)	36 (31.6%)	9 (19.1%)	27 (40.3%)	0.053
Coronary artery disease, *n* (%)	49 (43.0%)	19 (40.4%)	30 (44.8%)	0.898
Type of GLP-1 RA at initiation				
Exenatide, *n* (%)	5 (4.4%)	2 (4.3%)	3 (4.5)	0.987
Semaglutide, *n* (%)	78 (68.4%)	31 (66.0%)	47 (70.1%)	
Lixisenatide, *n* (%)	2 (1.8%)	1 (2.1%)	1 (1.5%)	
Dulaglutide, *n* (%)	25 (21.9%)	11 (23.4%)	14 (20.9)	
Liraglutide, *n* (%)	4 (3.5%)	2 (4.3%)	2 (3.0%)	

GLP-1 RA—glucagon-like peptide-1 receptor agonist; HbA1c—hemoglobin A1c. Continuous variables were compared using independent sample *t*-test and % using the chi-square test. Continuous variables are expressed as mean ± standard deviation.

## Data Availability

The data that supports the findings of the study are available from the corresponding author, upon reasonable request.

## Supplementary Materials

The following supporting information can be downloaded at: https://www.mdpi.com/article/10.3390/medicina61081343/s1, Figure S1: Mean HbA1c during follow-up; Figure S2: Mean BMI during follow-up.

## Abbreviations

The following abbreviations are used in this manuscript:

T2D	Type 2 diabetes
GLP-1 RAs	Glucagon-like peptide-1 receptor agonists
SGLT2	Sodium–glucose transporters 2
MACE	Major adverse cardiovascular event
HbA1c	Hemoglobin A1c
BMI	Body mass index
CKD	Chronic kidney disease
PAD	Peripheral arterial disease
CAD	Coronary artery disease
OADs	Oral antihyperglycemic drugs
