# Supplementary material for: Real-World Sex Differences in Response to Treatment with Glucagon-like Peptide-1 Receptor Agonists: Analysis of Single-Center Outpatient Case Series
Source: Medicina (Kaunas). 2025 Jul 25;61(8):1343. doi: 10.3390/medicina61081343 (PMC12388165; doi:10.3390/medicina61081343)
Supplement: Supplementary file 1 [file medicina-61-01343-s001.zip › medicina-3748531-supplementary.pdf]

Supplementary figures

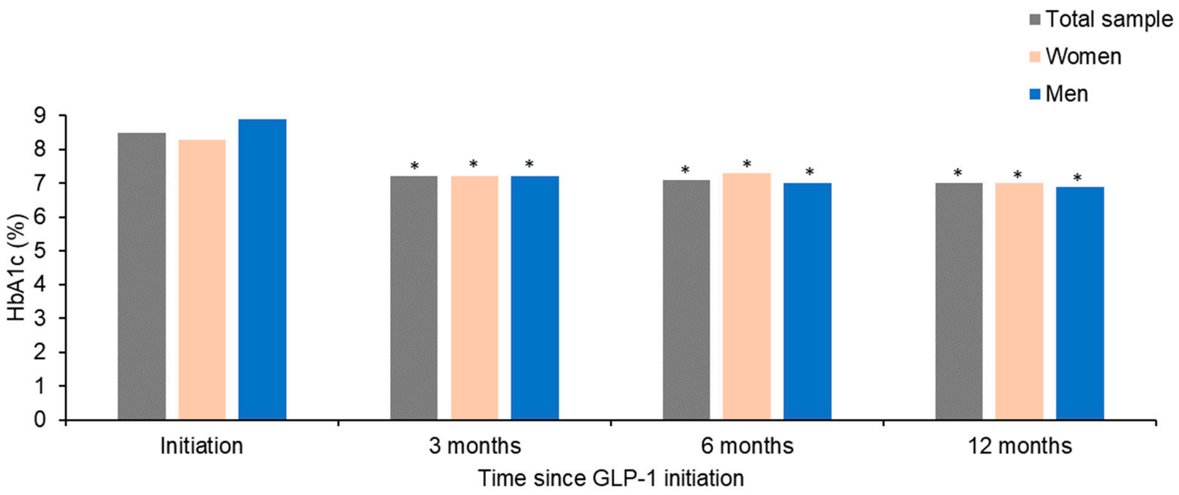

Supplementary Figure S1. Mean HbA1c during the follow-up

*Significant difference vs. GLP-1 initiation (p <0.001)

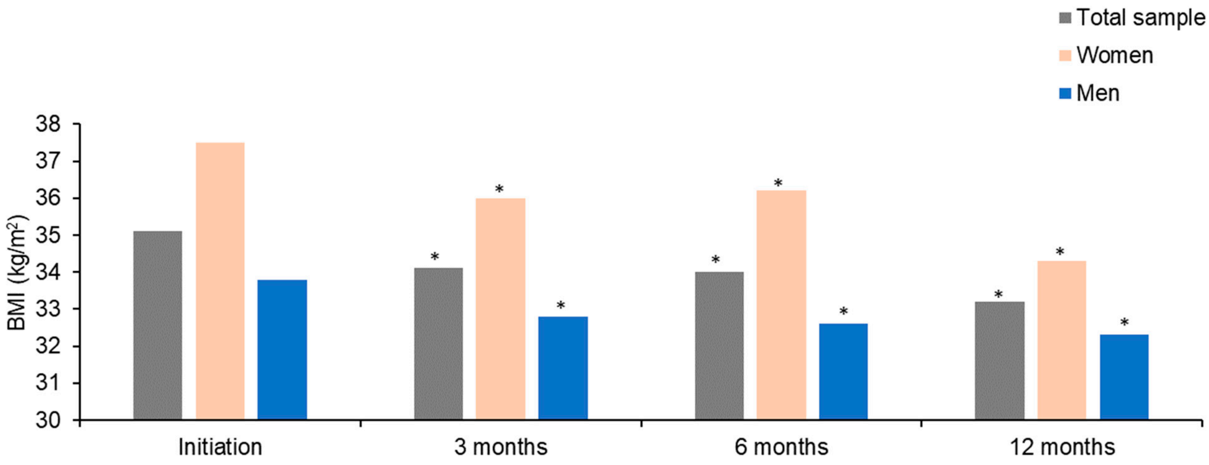

Supplementary Figure S2. Mean BMI during the follow-up
